# The association between serum uric acid / serum creatinine ratio and in-hospital outcomes in elderly patients with acute myocardial infarction

**DOI:** 10.1186/s12872-024-03720-6

**Published:** 2024-01-16

**Authors:** Lujing Jiang, JunGuo Jin, Xuyu He, Xiangming Hu, Lan Guo, Guo Chen, Yingling Zhou

**Affiliations:** 1 School of Medicine, South China University of Technology, Guangzhou, Guangdong 510006 China; 2 Guangdong Provincial People’s Hospital (Guangdong Academy of Medical Sciences), Southern Medical University, Guangzhou, Guangdong 510080 China; 3 Department of Cardiology, Guangdong Provincial People’s Hospital (Guangdong Academy of Medical Sciences, Southern Medical University, Guangzhou, Guangdong 510080 China

**Keywords:** Uric acid, SUA/Scr, Elderly patients, Acute myocardial infarction, In-hospital outcomes

## Abstract

**Background:**

The role of Serum uric acid (SUA) in acute myocardial infarction (AMI) was controversial, which might be influenced by the renal clearance function of the patients. The present study aimed to explore the association between serum uric acid to serum creatinine ratio (SUA/Scr), reflecting a net production of SUA, and the in-hospital outcomes of elderly patients with AMI.

**Methods:**

In this retrospective study, a total of 330 elderly AMI patients (≥ 75 years) were enrolled. Data of SUA and Scr on admission were collected to calculate SUA/Scr ratio. Logistic regression analysis and receiver-operating curves were performed to assess the association between SUA/Scr ratio and in-hospital major adverse cardiovascular events (MACEs) and all-cause death.

**Results:**

Among the 330 patients, 68 patients had MACEs and 44 patients died. Patients with MACEs or died had lower SUA/Scr values compared with those without MACEs or survival (*P* < 0.05). Univariate logistic analysis showed that a lower value of SUA/Scr (< 3.45) was significantly associated with in-hospital MACEs (odd ratios (OR): 2.359, 95% confidential interval (CI): 1.369–4.065, *P* = 0.002) and death (OR: 2.424, 95% CI: 1.275–4.608, *P* = 0.007). After correcting for confounding factors, a lower SUA/Scr value was still independently associated with in-hospital MACEs (OR: 2.144, 95% CI: 1.169–3.934, *P* = 0.014) and death (OR: 2.125, 95% CI: 1.050–4.302, *P* = 0.036). Subgroup analysis showed that the association between a lower SUA/Scr ratio and increased risk of in-hospital outcomes could observed only in males (OR: 2.511, 95%CI: 1.211–5.207, *P* = 0.013 for MACEs; OR: 2.730, 95% CI: 1.146–6.502, *P* = 0.023 for death).

**Conclusions:**

A lower SUA/Scr ratio was associated with an increased risk of in-hospital adverse events in elderly patients with AMI, especially in males, which maybe a marker of poor outcomes for elderly AMI patients.

**Supplementary Information:**

The online version contains supplementary material available at 10.1186/s12872-024-03720-6.

## Introduction

Despite notable advancements in reperfusion and revascularization strategies, as well as medicine therapy have contributed to a significant reduction in mortality rates after AMI in the past 10 years, AMI persists as a significant global cause of mortality, particularly among the elderly patients [1, 2]. It has been reported that nearly 1/3 of patients have AMI on admission, 2/3 of those who die from MI are aged > 75, and the morbidity and mortality rates of AMI are higher in elderly patients than in young adults [3, 4]. Therefore, it is of great significance to find new markers for more precise cardiovascular risk stratification in elderly patients with AMI.

Serum uric acid (SUA) is the end-product of purine metabolism, of which the serum level is determined by its production and subsequent renal excretion [5]. It is not only an indicator of renal function but is also related to many cardiovascular events, including AMI [6, 7]. However, conclusions of the effects of SUA on outcomes in elderly patients with AMI remain controversial [8–11], which may be due to the fact that some studies have overlooked the influence of renal function on SUA levels. Patients with poor renal function may have high SUA levels due to reduced renal excretion function of uric acid. Recently, the ratio of serum uric acid to serum creatinine (SUA/SCr), reflecting a net production of uric acid, has come into attention as a standardized SUA index of kidney function and a new marker for CVD [12]. It was reported that SUA/Scr might be a marker of poorer outcomes in patients with ischemic stroke [13]. Nevertheless, the influence of SUA/Scr on the outcomes of individuals with AMI, especially in elderly patients, remains unclear.

Thus, the present study aimed to explore the influence of SUA/SCr on in-hospital outcomes in elderly patients with AMI.

## Methods

### Study population

This study was a single-center, retrospective study and approved by the Institutional Review Board of Guangdong Provincial People’s Hospital (No. GDREC2016411H(R1)). We included patients who were diagnosed with AMI, including both ST-segment elevation MI (STEMI) and non-ST-segment elevation MI (NSTEMI), on admission to Guangdong Provincial People’s Hospital and were aged 75 years or older from January 2015 to December 2020. And we excluded patients with missing SUA and/or Scr values. The study was conducted in accordance with the principles of the Declaration of Helsinki.

### Data collection

Demographic information and medical history, including hypertension, diabetes, MI, prior coronary revascularization, stroke, information on medications before and during admission, and the percentage of very old patients were collected from medical records by three experienced data inspectors [14]. The type of AMI, STEMI or NSTEMI, was recorded. Additionally, the TIMI risk scores on admission [15, 16], symptom-onset-to-balloon time, information about multi-vessel disease, cardiac arrest before admission, details of percutaneous coronary intervention (PCI), as well as left ventricular ejection fraction (LVEF) of the index hospitalization were documented.

The first available laboratory data at admission were collected including peak creatine kinase myocardial band (CK-MB), hemoglobin (Hb), total bilirubin, total cholesterol, high-density lipoprotein cholesterol (HDL-c), low-density lipoprotein cholesterol (LDL-c), N-terminal pro-brain natriuretic peptide (NT-proBNP), serum uric acid (SUA), and serum creatinine (Scr). SUA/Scr was calculated as SUA (umol/L) divided by Scr (umol/L) [17]. All blood samples were analyzed using standard examination methods in the laboratory of our hospital, adhering to the ISO 9000 Quality Management and Assurance Standards at the medical center.

### Outcomes

Major adverse cardiovascular events (MACEs) in hospital, which comprise a composite outcome of all-cause death, major bleeding, ischemic stroke, mechanical complication of AMI, cardiogenic shock, and re-infarction was the primary endpoint of the investigation. Major bleeding was defined as a decrease in hemoglobin level of at least 5 g/dL, cardiac tamponade, any intracranial hemorrhage or gastrointestinal bleeding based on the Bleeding Academic Research Consortium (BARC) definition [18]. Mechanical complication included in-hospital rupture of the ventricular free wall, rupture of the papillary muscle, and ventricular septal rupture. Cardiogenic shock was only for patients who were not in shock on admission [3]. Re-infarction was defined based on the universal definition of myocardial infarction guidelines [19]. Briefly, re-infarction was diagnosed as an AMI occurring within 28 days of an incident MI that complied with the Fourth Universal Definition of Myocardial Infarction [19]. For patients who experienced several in-hospital MACE events, only one event was counted in the calculation of MACEs. In other words, for patient who died finally, only one event was counted no matter how many cardiovascular events he experienced when he was alive during hospitalization. For patients who did not die during hospitalization, only the first occurrence of cardiovascular event was counted.

### Statistical analysis

In the study, continuous variables were showed as means ± standard deviations or medians (interquartile range), and categorical variables were represented by frequencies (percentages). *t*-test or Mann-Whitney U test were performed to compare continuous variables and Chi-square or Fisher’s exact tests were performed to compare categorical variables. Univariate logistic regression analysis was conducted in order to assess the correlation between clinical variables and in-hospital outcomes. Multivariate regression analysis was further conducted using a method of forward stepwise regression for variables of significance in univariate analysis and other clinically important factors.

The statistical analysis was conducted using SPSS version 26.0 (SPSS, Inc., Chicago, IL, USA) and R (version 2.4.3). *P* < 0.05 (two-sided) was considered statistically significant.

## Results

### Baseline characteristics

A total of 2,404 patients were diagnosed with AMI, and 365 patients of whom were aged ≥ 75 years. After excluding 35 patients who lacked SUA and/or Scr values, a final sample of 330 elderly AMI patients was included. Table 1 showed the baseline characteristics of the 330 elderly AMI patients. Among these patients, 68 had MACEs and 44 died during hospitalization, and 54.2% of them were aged ≥ 80 years. Patients who experienced MACEs exhibited a significantly lower value of SUA/Scr compared to those did not experience MACEs (*P* = 0.001). Furthermore, in comparison to those without MACEs, patients with MACEs lower LDL-c (*P* = 0.008), lower LVEF (*P* < 0.001), higher TIMI risk score (*P* < 0.001), higher creatinine (*P* < 0.001) and a longer symptom-onset-to-balloon time (*P* = 0.029), and were more likely to take diuretics and angiotensin receptor antagonist (ARB) before and during hospitalization (*P* = 0.001 for history of diuretics, *P* = 0.029 for history of ARB, *P* = 0.002 for diuretics during hospitalization and *P* < 0.001 for ARB during hospitalization) but less likely to receive PCI (*P* = 0.001).

**Table 1:** The baseline characteristics of elderly patients with AMI.

Variable	Overall (*n* = 330)	MACEs (*n* = 68)	Non-MACEs (*n* = 262)	*P*-Value	Death (*n* = 44)	Survival (*n* = 286)	*P*-Value
Age (years)	80.0 (77.0, 83.0)	81.0 (78.0, 84.0)	80.0 (77.0, 83.0)	0.193	81.0 (79.0, 83.0)	80.0 (77.0, 83.0)	0.162
≥ 80 (n, %)	180 (54.5)	40 (58.9)	140 (54.2)	0.427	28 (63.6)	152 (53.1)	0.193
Male (n, %)	217 (65.8)	47 (69.2)	170 (64.8)	0.512	30 (68.2)	187 (65.4)	0.716
Medical history (n, %)							
Hypertension	223 (67.6)	48 (70.6)	175 (66.8)	0.551	31 (70.5)	192 (67.1)	0.661
Diabetes	100 (30.3)	25 (36.8)	75 (28.6)	0.193	15 (34.1)	85 (29.7)	0.557
Prior MI	46 (13.9)	11 (16.2)	35 (13.4)	0.550	8 (18.2)	38 (13.3)	0.383
Prior PCI or CABG	49 (14.8)	10 (14.7)	39 (14.9)	0.970	6 (13.6)	43 (15.0)	0.808
Prior stroke	45 (13.6)	8 (11.8)	37 (14.1)	0.614	3 (6.8)	42 (14.7)	0.157
Medication history (n, %)							
Diuretics	57 (17.2)	21 (30.9)	36 (13.7)	0.001^*^	17 (38.6)	40 (14.0)	< 0.001^*^
Dapagliflozin	11 (3.3)	1 (1.5)	10 (3.8)	0.561	0 (0)	11 (3.8)	0.371
Allopurinol	5 (1.5)	2 (2.9)	3 (1.1)	0.601	2 (4.5)	3 (1.0)	0.269
Febuxostat	4 (1.2)	1 (1.5)	3 (1.1)	1.000	0 (0)	4 (1.4)	1.000
ARB	70 (21.2)	21 (30.9)	49 (18.7)	0.029^*^	13 (29.5)	57 (19.9)	0.146
Cardiac arrest before admission	23 (7.0)	5 (7.4)	18 (6.9)	0.889	4 (9.1)	19 (6.6)	0.553
Current smoking	59 (17.9)	10 (14.7)	49 (18.7)	0.443	5 (11.4)	54 (18.9)	0.226
Clinical characteristics (n, %)							
STEMI	235 (71.2)	51 (75.0)	184 (70.2)	0.439	32 (72.7)	203 (71.0)	0.812
TIMI risk score	4.0 (3.0, 6.0)	5.0 (4.0, 7.8)	4.0 (3.0, 5.0)	< 0.001^*^	5.5 (4.0, 8.0)	4.0 (3.0, 5.0)	< 0.001^*^
Revascularization details (n, %)							
Multi-vessel disease	238 (72.1)	49 (72.1)	189 (72.1)	0.990	31 (70.5)	207 (72.4)	0.791
Symptom-onset-to-balloon time within 12 h	170 (51.5)	27 (39.7)	143 (54.6)	0.029^*^	16 (36.4)	154 (53.8)	0.031^*^
PCI or not	291 (88.2)	52 (76.5)	239 (91.2)	0.001^*^	33 (75.0)	258 (90.2)	0.004^*^
Complete revascularization	208 (63.0)	39 (57.4)	169 (64.5)	0.276	25 (56.8)	183 (64.0)	0.359
Medication during hospitalization (n, %)							
Diuretics	186 (55.8)	49 (72.1)	137 (52.3)	0.002^*^	33 (75.0)	153 (53.5)	0.006^*^
Dapagliflozin	3 (0.9)	1 (1.5)	2 (7.6)	1.000	1 (2.3)	2 (0.7)	0.865
Allopurinol	1 (0.3)	0 (0)	1 (3.8)	1.000	0 (0)	1 (0.3)	1.000
Febuxostat	1 (0.3)	1 (1.5)	0 (0)	0.467	0 (0)	1 (0.3)	1.000
ARB	40 (12.1)	12 (17.6)	28 (10.7)	< 0.001^*^	8 (18.2)	32 (11.2)	0.186
Dual anti-platelet therapy	330 (100)	68 (100)	262 (100)	1.000	44 (100)	286 (100)	1.000
Statins	328 (99.4)	67 (98.5)	261 (99.6)	0.212	43 (97.7)	285 (99.7)	0.626
Biochemical variables							
Peak CK-MB	138.5 (60.4, 276.4)	172.5 (61.7, 307.7)	134.0 (59.9, 270.1)	0.229	200.3 (77.3, 341.7)	132.6 (58.6, 270.0)	0.063
Hemoglobin (g/L)	127.0 (113.0, 137.0)	122.0 (104.3, 136.8)	127.5 (115.0, 138.0)	0.117	124.0 (103.3, 135.9)	127.0 (114.4, 138.0)	0.305
Total bilirubin (mmol/L)	14.9 (10.8, 19.8)	17.6 (12.0,21.9)	14.7 (10.7, 18.6)	0.068	17.9 (12.2, 21.9)	14.8 (10.7,19.2)	0.077
Total cholesterol (mmol/L)	4.5 (3.8, 5.2)	4.2 (3.7, 5.1)	4.5 (3.9, 5.2)	0.112	4.2 (3.6, 5.2)	4.5 (3.9, 5.2)	0.076
HDL-c (mmol/L)	1.0 (0.9, 1.2)	1.0 (0.9, 1.3)	1.0 (0.9, 1.2)	0.907	1.1 (0.9, 1.3)	1.0 (0.9, 1.2)	0.223
LDL-c (mmol/L)	2.8 (2.3, 3.4)	2.6 (2.1, 3.1)	2.9 (2.4, 3.5)	0.008^*^	2.5 (2.0, 2.9)	2.9 (2.4, 3.5)	0.005^*^
Uric acid (µmol/L)	419.1 (342.8, 515.1)	419.1 (360.5, 555.8)	419.5 (341.8, 504.9)	0.337	414.1 (377.1, 551.0)	419.1 (341.5, 512.0)	0.307
Creatinine (µmol/L)	98.0 (78.0, 126.9)	122.3 (98.1, 147.8)	92.6 (75.8, 121.0)	< 0.001^*^	125.9 (98.5, 149.5)	94.0 (76.1, 123.0)	< 0.001^*^
SUA/Scr	4.2 (3.0, 5.8)	3.5 (2.6, 4.8)	4.5 (3.2, 5.9)	0.001^*^	3.4 (2.6, 4.9)	4.4 (3.1, 5.9)	0.016^*^
NT-pro BNP (pg/ml)	4202.5 (1680.5, 8605.0)	4455.5 (2905.5, 9025.3)	4069.0 (1468.3, 8443.5)	0.078	4175.5 (2820.3, 8618.8)	4200.0 (1535.0, 8595.0)	0.327
LVEF (%)	47.0 (38.0, 58.0)	38.0 (33.0, 47.8)	49.0 (40.0, 59.3)	< 0.001^*^	37.5 (32.0, 46.0)	48.0 (40.0, 59.0)	< 0.001^*^

ARB, angiotensin receptor antagonist; STEMI, ST-segment elevation MI; PCI, percutaneous coronary intervention; CK-MB, creatine kinase myocardial band; HDL-c, high density lipoprotein cholesterol; LDL-c, low density lipoprotein cholesterol; SUA/Scr, serum uric acid/ serum creatinine; NT-pro BNP, N-terminal pro-brain natriuretic peptide; LVEF, left ventricular ejection fraction. * *P* < 0.05

Among the study participants, those who died during hospitalization also exhibited lower value of SUA/Scr (*P* = 0.016), lower LDL-c level (*P* = 0.005), lower LVEF (*P* < 0.001), higher creatinine (*P* < 0.001), higher TIMI risk scores (*P* < 0.001) and a longer symptom-onset-to-balloon time (*P* = 0.031), and were more likely to take diuretics before and during hospitalization (*P* = 0.020 and *P* = 0.006, respectively) but less likely to receive PCI (*P* = 0.004) compared to those who were survived.

### ROC analysis

**Fig. 1:**
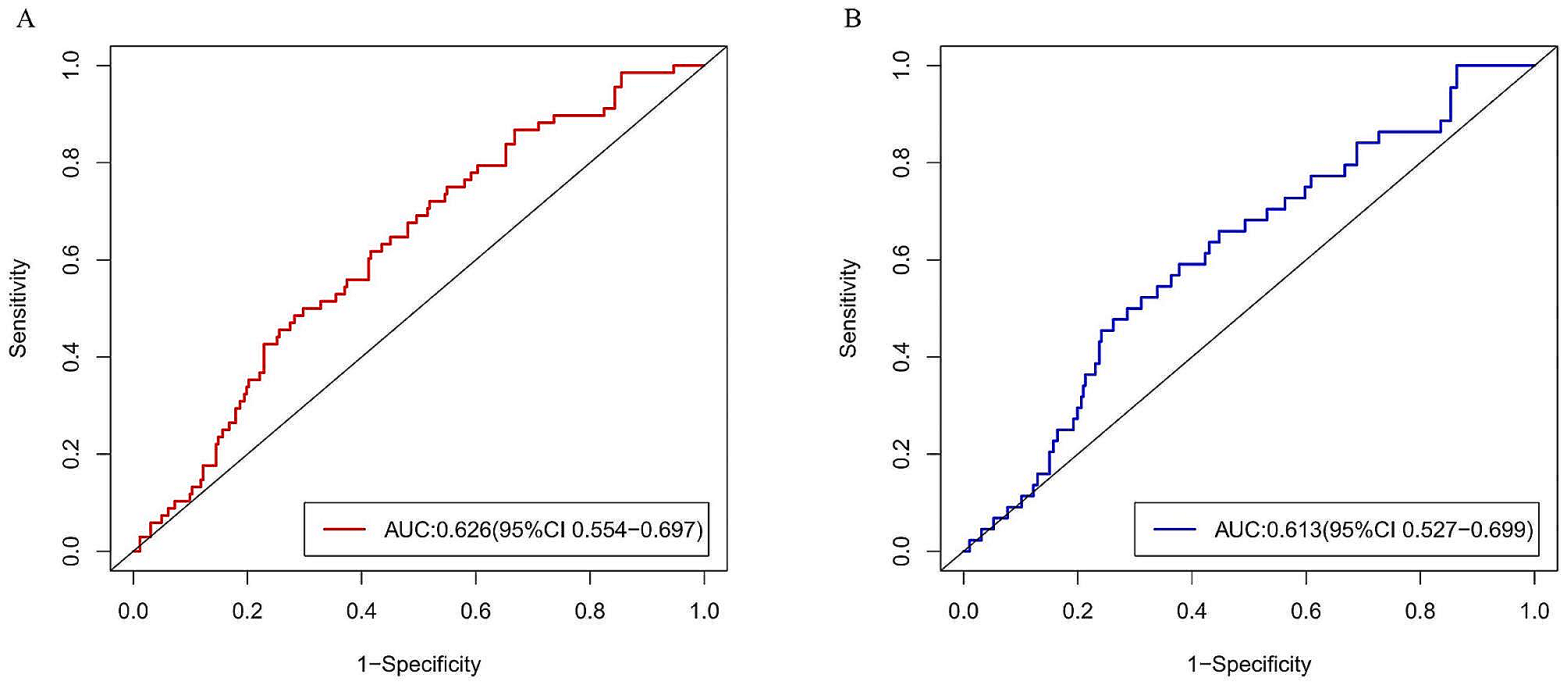
ROC curve for in-hospital MACEs and death. **(A)** ROC curve for in-hospital MACEs. **(B)** ROC curve for in-hospital death. AUC, area under curve; CI, confidence incidence

ROC curve analysis indicated that the optimal cut-off values of SUA/Scr for in-hospital MACEs and all-cause death in elderly AMI patients was 3.41 and 3.23, respectively (Fig. 1A and B). To establish a signal value for clinical diagnosis, a value of 3.45 was ultimately decided. Then patients were divided into low SUA/Scr group (SUA/Scr < 3.45) and high SUA/Scr group (SUA/Scr ≥ 3.45). The incidences of in-hospital MACEs and all cause death of the two groups were exhibited in Table 2. Comparatively, elderly patients with a low SUA/Scr ratio exhibited a significant increase in the incidence of in-hospital MACEs and death when compared to those with a high SUA/Scr ratio (*P* = 0.002 for MACEs, *P* = 0.006 for death) (Table 2 and Supplementary Table 1).

**Table 2:** In-hospital outcomes in low and high SUA/Scr groups

In-hospital outcomes	Low SUA/Scr < 3.45(*n* = 112)	high SUA/Scr ≧ 3.45 (*n* = 218)	*P*-Value
MACEs	34 (30.4%)	34 (15.6%)	0.002^*^
All-cause death	23 (20.5%)	21 (9.6%)	0.006^*^
Mechanical complications of MI	6 (5.4%)	6 (2.8%)	
Rupture of the ventricular free wall	2	4	
Rupture of the papillary muscle	1	2	
Ventricular septal rupture	3	0	
Cardiogenic shock	6	7	
Malignant arrhythmia	5	4	
Pericardial tamponade	2	2	
Mesenteric embolism	0	1	
Pulmonary embolism	1	0	
Major bleeding	1	0	
Infectious shock	2	1	
Cardiogenic shock	9 (8.0%)	6 (2.8%)	
Reinfarction	0 (0)	1 (0.5%)	
Major bleeding	2 (1.8%)	6 (2.8%)	
Cardiac tamponade	0	0	
Gastrointestinal bleeding	1	5	
Access site bleeding	1	1	

MACEs: major adverse cardiovascular events; * *P* < 0.05

### Logistic regression analysis

**Fig. 2:**
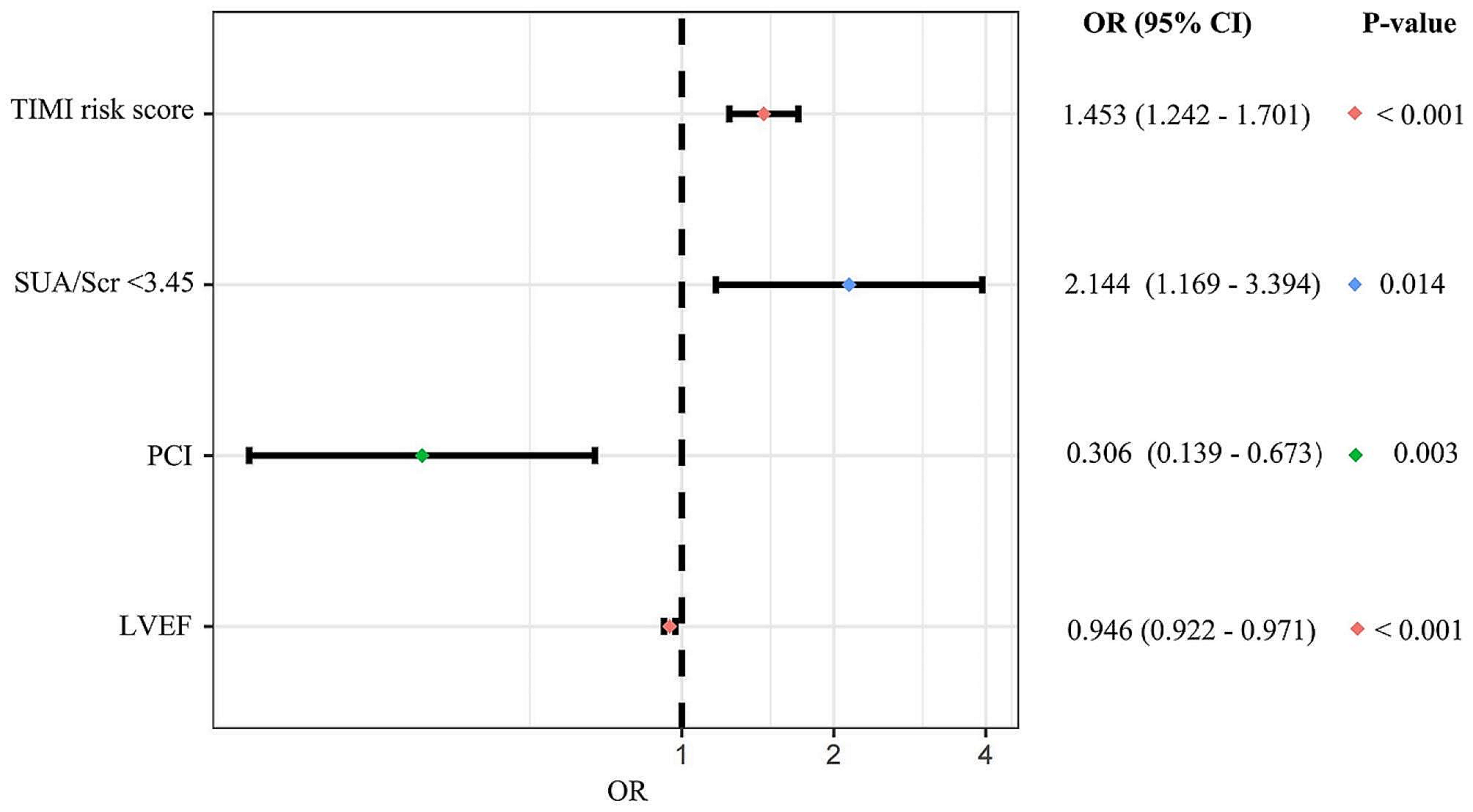
Forest plot for in-hospital MACEs. Forest plots showing risk factors for in-hospital MACEs by multivariate logistic analysis. SUA/Scr, serum uric acid/serum creatinine; PCI, percutaneous coronary intervention; LVEF, left ventricular ejection fraction; OR, odd ratios; CI, confidence incidence

**Fig. 3:**
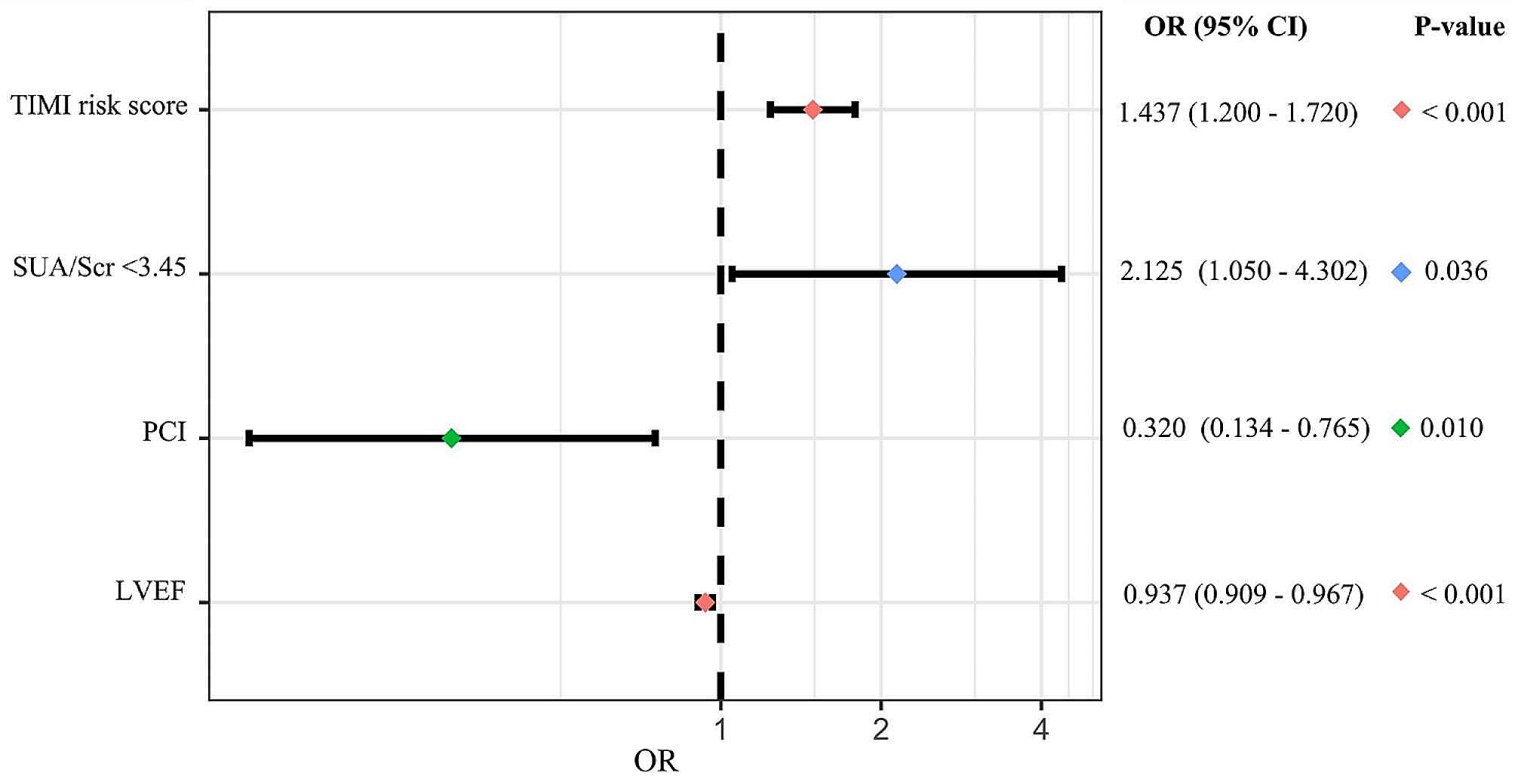
Forest plot for in-hospital death. Forest plots showing risk factors for in-hospital death by multivariate logistic analysis. SUA/Scr, serum uric acid/serum creatinine; PCI, percutaneous coronary intervention; LVEF, left ventricular ejection fraction; OR, odd ratios; CI, confidence incidence

In the univariate logistic analysis, history of diuretics ( OR: 2.805, 95%CI: 1.504–5.23, *P* = 0.001), history of ARB ( OR: 1.942, 95%CI: 1.065–3.542, *P* = 0.030), diuretics during hospitalization (OR: 2.353, 95%CI: 1.314–4.213, *P* = 0.004), symptom-onset-to-balloon time within 12 h (OR: 0.548, 95%CI: 0.318–0.943, *P* = 0.030), undergoing PCI (OR: 0.313, 95%CI: 0.155–0.633, *P* = 0.001), LVEF (OR: 0.942, 95%CI: 0.920–0.966, *P* < 0.001), TIMI risk score (OR: 1.461, 95%CI: 1.262–1.691, *P* < 0.001), LDL-c (OR: 0.670, 95%CI: 0.473–0.947, *P* = 0.023) and SUA/Scr < 3.45 (OR: 2.359, 95%CI: 1.369–4.065, *P* = 0.002) were significantly associated with in-hospital MACEs (Table 3). After adjusting for confounder shown in Table 3, SUA/Scr < 3.45 was still independently related to MACEs (OR: 2.144, 95%CI: 1.169–3.934, *P* = 0.014) (Table 3, Supplementary Table 2, Fig. 2) in the multivariable analysis. Similarly, the multivariable analysis suggested that after adjusting for the confounding factors in Table 4, SUA/Scr < 3.45 was also significantly associated with all-cause death during hospitalization (OR: 2.125, 95%CI: 1.050–4.302, *P* = 0.036) (Table 4, Supplementary Table 2, Fig. 3).

**Table 3:** Univariate and multivariate analysis for in-hospital MACEs

Variable	Univariate analysis		Multivariate analysis ^a^	
	Odd ratios (95%CI)	*P*-Value	Odd ratios (95% CI)	*P*-Value
Age	1.042 (0.978–1.109)	0.201		
Male	1.211 (0.683–2.149)	0.513		
Hypertension	1.193 (0.667–2.134)	0.552		
Diabetes	1.450 (0.827–2.540)	0.195		
Smoking status	0.749 (0.358–1.570)	0.445		
Multi-vessel disease	0.996 (0.550–1.805)	0.990		
Complete revascularization	0.740 (0.430–1.274)	0.277		
History of diuretics	2.805 (1.504–5.231)	0.001^*^		
History of ARB	1.942 (1.065–3.542)	0.030^*^		
Diuretics during hospitalization	2.353 (1.314–4.213)	0.004^*^		
ARB during hospitalization	1.791 (0.857–3.740)	0.121		
Symptom-onset-to-balloon time within 12 h	0.548 (0.318–0.943)	0.030^*^		
PCI	0.313 (0.155–0.633)	0.001^*^	0.306 (0.139–0.673)	0.003^*^
STEMI or NSTEMI	1.272 (0.691–2.339)	0.439		
LVEF	0.942 (0.920–0.966)	< 0.001^*^	0.946 (0.922–0.971)	< 0.001^*^
TIMI risk score	1.461 (1.262–1.691)	< 0.001^*^	1.453 (1.242–1.701)	< 0.001^*^
LDL-c	0.670 (0.473–0.947)	0.023^*^		
SUA/Scr < 3.45	2.359 (1.369–4.065)	0.002^*^	2.144 (1.169–3.934)	0.014^*^

^a^ Multivariate analysis: adjusting for age, gender, multi-vessel disease, history of diuretics, history of ARB, diuretics during hospitalization, PCI or not, smoking status, LVEF and TIMI risk score

MACEs: major adverse cardiovascular events; CI, confidence interval; ARB, angiotensin receptor antagonist; PCI: percutaneous coronary intervention; LVEF: left ventricular ejection fraction; STEMI: ST-segment elevation myocardial infarction; LDL-c, low density lipoprotein cholesterol; SUA/Scr: serum uric acid/serum creatinine. * *P* < 0.05

**Table 4:** Univariate and multivariate analysis for in-hospital death

Variable	Univariate analysis		Multivariate analysis ^a^	
	Odd ratios (95% CI)	*P*-Value	Odd ratios (95% CI)	*P*-Value
Age	1.046 (0.971–1.126)	0.237		
Male	1.134 (0.575–2.238)	0.716		
Hypertension	1.167 (0.584–2.335)	0.661		
Diabetes	1.223 (0.624–2.397)	0.557		
Smoking status	0.551 (0.207–1.463)	0.232		
Multi-vessel disease	0.910 (0.453–1.828)	0.791		
History of diuretics	3.872 (1.937–7.741)	< 0.001^*^		
History of ARB	1.685 (0.829–3.426)	0.150		
Diuretics during hospitalization	2.682 (1.305–5.515)	0.007^*^		
ARB during hospitalization	1.764 (0.754–4.126)	0.191		
Symptom-onset-to-balloon time witin12hours	0.490 (0.254–0.945)	0.033^*^		
Complete revascularization	0.741 (0.389–1.409)	0.360		
PCI	0.326 (0.148–0.714)	0.005^*^	0.320 (0.134–0.765)	0.010^*^
STEMI	1.090 (0.536–2.220)	0.812		
LVEF	0.933 (0.906–0.962)	< 0.001^*^	0.937 (0.909–0.967)	< 0.001^*^
TIMI risk score	1.442 (1.221–1.702)	< 0.001^*^	1.437 (1.200–1.720)	< 0.001^*^
LDL-c	0.573 (0.372–0.884)	0.012^*^		
SUA/Scr < 3.45	2.424 (1.275–4.608)	0.007^*^	2.125 (1.050–4.302)	0.036^*^

^a^ Multivariate analysis: adjusting for age, gender, multi-vessel disease, history of diuretics, diuretics during hospitalization, PCI or not, smoking status, LVEF and TIMI risk score

CI, confidence interval; ARB, angiotensin receptor antagonist; PCI: percutaneous coronary intervention; STEMI: ST-segment elevation myocardial infarction; LVEF: left ventricular ejection fraction; LDL-c, low density lipoprotein cholesterol; SUA/Scr: serum uric acid/serum creatinine. * *P* < 0.05

### The results of subgroup analysis

Given that previous studies have indicated that the relationship between SUA and CVD may be influenced by gender, we conducted a subgroup analysis to examine whether gender could influence the value of SUA/Scr in the outcomes among elderly AMI patients during hospitalization [20, 21]. Interesting, the results showed that the association between a lower SUA/Scr value (< 3.45) and in hospital outcomes was observed only in males, with an OR of 2.511 (95% CI: 1.211–5.207, *P* = 0.013) for in-hospital MACEs and an OR of 2.730 (95% CI: 1.146–6.502, *P* = 0.023) for all cause death during hospitalization in the multivariable analysis (Table 5).

**Table 5:** Subgroup analysis based on gender

Subgroup	SUA/Scr < 3.45	SUA/Scr $\underline{\underline > }$ 3.45	Univariate analysis			Multivariate analysis ^a^		
	(No. of events/patients)		Odd ratios (95% CI)	*P-*value	*P* for interaction	Odd ratios (95% CI)	*P*-value	*P* for interaction
MACEs								
Male	25/81	22/136	2.313 (1.200-4.459)	0.012^*^	0.959	2.511 (1.211–5.207)	0.013^b^	0.208
Female	9/31	12/82	2.386 (0.888–6.410)	0.084				
Deaths								
Male	17/81	13/136	2.513 (1.149–5.498)	0.021^*^	0.861	2.730 (1.146–6.502)	0.023^b^	0.165
Female	6/31	8/82	2.220 (0.702–7.022)	0.175				

^a^ Forward stepwise regression method was used in the multivariate logistic analysis

^b^ Adjusted for age, smoking status, multi vessel disease, TIMI risk score, PCI or not, Symptom-onset-to-balloon time within 12 h, complete revascularization, LVEF, hypertension, DM, type of AMI.

SUA/Scr, serum uric acid/serum creatinine; MACEs: major adverse cardiovascular events. * *P* < 0.05

## Discussion

In the present study, we found that a lower value of renal function normalized SUA, SUA/Scr, was significantly related to an increased risk of in-hospital adverse outcomes, including MACEs and death, among elderly AMI patients. Moreover, subgroup analysis revealed that this relationship was more meaningful in males. As far as we know, this is the first study to explore the relationship between SUA/Scr and in-hospital outcome in elderly AMI patients.

In recent years, the role of SUA levels in AMI has remained controversial and studies in elderly patients have been limited [22–29]. Some studies have suggested that SUA was an independent risk factor for AMI, while others have held the opposite voice [9, 28, 29]. Li et al. have reported that high SUA level was an independent risk factor in elderly patients with STEMI [9] and two Italian studies found that high admission levels of SUA were independently associated with in-hospital adverse outcomes and mortality in patients with acute coronary syndrome [26, 27]. However, Liu et al. have found no relationship between high SUA levels and mortality of STEMI patients with Killip classes II-IV [28]. A cohort study of 375,163 participants has suggested that both low and high SUA levels have an association with increased mortality of CVD, suggested a U-shaped correlation between SUA and adverse outcomes of CVD [29]. The conflicting conclusions might be due to variations in age groups among the study subjects, with the previous evidence being more prevalent in younger patients than in older patients [22, 23]. Additionally, SUA levels are influenced by renal clearance function, and higher SUA levels could be observed in patients with poor renal function [30]. Therefore, SUA/Scr, which reflects renal function-normalized SUA, could serve as a more accurate indicator of AMI.

Previous studies have explored the value of SUA/Scr ratio in some specific clinical outcomes, but less in the field of CVD [31–34]. Chen et al. have identified SUA/Scr as an independent risk factor for diabetic kidney disease in patients with type 2 diabetes mellitus, while there was no link between SUA/Scr and macrovascular disease [33]. A recent study has reported that evaluated SUA/Scr ratio was negatively associated with HbA1c in patients with diabetes [34]. Gong et al. have suggested that among patients with acute ischemic stroke, a lower SUA/Scr value was related to poor functional outcomes [13]. Our study added to this knowledge by revealing that a lower SUA/Scr ratio was associated with adverse in-hospital outcomes in elderly AMI patients, including MACEs and all cause death. And this relationship remained significant after correcting for various confounding factors, including age, gender, multi-vessel disease, symptom-to-onset-time, PCI status, medications before and during hospitalization, smoking, LVEF, type of AMI, and TIMI risk score. Recently, in the Uric Acid Right for Heart Health (URRAH) study based on Italian community general population, Casiglia et al. found that higher SUA/Scr was an independent risk of cardiovascular events with a cut-off value of 5.35 (mg/dl) [35]. Meanwhile, our study found that lower SUA/Scr value with a cut-off of 3.45 µmol/L (5.12 mg/dL) was associated with in-hospital MACEs and all-cause death among Chinese elderly patients with AMI. The possible reason for the inverse association between SUA/Scr and cardiovascular risk might be that SUA may exert anti-oxidant properties to scavenge reactive oxygen species in acute stress instead of a chronic pro-oxidative role in general population [36]. Besides, the different ethnicity, age and disease status of the study population may also contribute to the different cut-off values.

It is interesting that in the present study, patients with adverse outcomes showed similar SUA at admission with patients without events. The higher incidence of diuretics and ARB use in patients with adverse outcomes may explain this lack of difference. To overtake this issue, we added diuretic use to the multivariable model, and found the use of diuretic had no effect on the association between SUA/Scr and in-hospital outcomes in the elderly AMI patients. This result was similar to that of another analysis of the URRAH study which showed that individuals with hyperuricemia and diuretic use exhibited a similar risk compared with those presented hyperuricemia in absence of diuretic therapy [37]. On the other hand, we found that timely PCI in elderly patients with AMI was beneficial in improving in-hospital outcomes for the index AMI after adjustments for potential confounders. This was consistent with growing evidence that primary PCI for STEMI and early invasive strategy for NSTEMI had prognostic benefits regardless of age, indicating a more positive PCI strategy should also be adopted in elderly patients, which was not always applied in clinical practice [38–40].

The present study also observed a gender-related differences in the association between SUA/Scr and in-hospital outcomes. Previous studies also have reported that the relationship between SUA and CVD was influenced by gender, while the conclusions seem to be inconsistent [41–43]. Yuan et al. have found that SUA was U-shapedly associated with cardiovascular outcomes in males without considering kidney function [42]. Kang et al. have reported that among 27,490 participants older than 40 years with normal kidney function, a significant association between lower SUA levels (≤ 4.0 mg/dl) and all-cause mortality, including cardiovascular mortality, was observed only in men, but not in women [43]. In the present study, we observed a stronger association between renal function normalized SUA (SUA/Scr) and in-hospital outcomes among elderly male patients with AMI, not in women. It could be explained by that the uricosuric effect in elderly women, who have lower levels of estrogen, decreased and the net level of SUA was higher than men [44]. Therefore, the above conclusion was more significant in males.

There were several limitations in the present study. One of the major limitations of this study is its retrospective and observational nature. Thus, direction of the observed relationship could not be determined. It could be that SUA exert excellent antioxidant for the infarcted heart, but it could also be that serum UA levels were influenced by severity of the index AMI. Additionally, this study was conducted at a single center and the sample size was limited, which might cause selection bias. The present study was conducted on Chinese elderly patients with AMI only and the applicability of the findings to other populations needs to be further explored [45]. Lastly, the influence of baseline renal function could not be excluded in the present study. In order to further comprehensively assess the value of SUA/Scr, future studies with larger sample size, careful stratification and long-term follow-up are needed.

## Conclusions

The present study suggests that a lower SUA/Scr ratio is associated with an increased risk of in-hospital adverse events in elderly AMI patients. This association is significantly observed in males, but not in females. SUA/Scr is an inexpensive and effective index, and could be used as an effective biomarker for elderly AMI patients. Future prospective studies with larger sample sizes are needed to validate these results and to better understand the potential clinical implications of SUA/Scr in this patient population.

### Electronic supplementary material

Below is the link to the electronic supplementary material.

Supplementary Material 1

## Data Availability

The data of the study can be available from the corresponding author.

## Abbreviations

AMI: acute myocardial infarction
SUA: serum uric acid
Scr: serum creatinine
SUA/Scr: the ratio of serum uric acid to serum creatinine
CVD: cardiovascular disease
STEMI: ST-segment elevation myocardial infarction
NSTEMI: non-ST-segment myocardial infarction
LVEF: left ventricular ejection fraction
CK-MB: peak creatine kinase myocardial band
Hb: hemoglobin
HDL: high-density lipoprotein cholesterol
LDL: low-density lipoprotein cholesterol
NT-pro-BNP: N-terminal pro-brain natriuretic peptide
MACEs: major adverse cardiovascular events
ARB: angiotensin receptor antagonist
