# Supplementary material for: The association between serum uric acid / serum creatinine ratio and in-hospital outcomes in elderly patients with acute myocardial infarction
Source: BMC Cardiovasc Disord. 2024 Jan 16;24:52. doi: 10.1186/s12872-024-03720-6 (PMC10792824; doi:10.1186/s12872-024-03720-6)
Supplement: Supplementary file 1 — Supplementary Material 1 [file 12872_2024_3720_MOESM1_ESM.docx]

**Supplementary Table 1. In-hospital outcomes in low and high SUA/Scr groups**

| **In-hospital outcomes** | **Low SUA/Scr**  **< 3.45(n = 112)** | **high SUA/Scr**  **≧ 3.45 (n = 218)** | ***P*-Value** |
| --- | --- | --- | --- |
| **MACEs (n, %)** | 34 (30.4%) | 34 (15.6%) | 0.002^*^ |
| **Non-death MACEs (n, %)** | 11 (9.8%) | 13 (6.0%) | 0.163 |
| Ischemic MACEs | 0 (0) | 1 (0.5%) | 1.000 |
| Reinfarction | 0 | 1 |  |
| Heart failure-related MACEs | 9 (8.0%) | 6 (2.8%) | 0.028^*^ |
| Cardiogenic shock | 9 | 6 |  |
| Major bleeding | 2 (1.8%) | 6 (2.8%) | 0.871 |
| Cardiac tamponade | 0 | 0 |  |
| Gastrointestinal bleeding | 1 | 5 |  |
| Access site bleeding | 1 | 1 |  |
| **All-cause death (n, %)** | 23 (20.5%) | 21 (9.6%) | 0.006^*^ |
| Ischemic MACEs | 1 (0.9%) | 1 (0.5%) | 1.000 |
| Mesenteric embolism | 0 | 1 |  |
| Pulmonary embolism | 1 | 0 |  |
| Heart failure-related MACEs | 19 (17.0%) | 19 (8.7%) | 0.026^*^ |
| Mechanical complications of MI | 6 | 6 |  |
| Rupture of the ventricular free wall | 2 | 4 |  |
| Rupture of the papillary muscle | 1 | 2 |  |
| Ventricular septal rupture | 3 | 0 |  |
| Cardiogenic shock | 6 | 7 |  |
| Malignant arrhythmia | 5 | 4 |  |
| Pericardial tamponade | 2 | 2 |  |
| Major bleeding | 1 (0.9%) | 0 (0) | 0.734 |
| Others | 2 (1.8%) | 1 (0.5%) | 0.555 |

MACEs, major adverse cardiovascular events; MI, myocardial infarction; * *P* < 0.05

**Supplementary Table 2. Lower SUA/Scr was an independent predictor for adverse clinical outcomes by adjusting diuretics**

|  | **Odd ratios (95% CI)** | ***P*-value*** |
| --- | --- | --- |
| **Model 1 for MACEs** |  |  |
| SUA/Scr <3.45 | 2.224 (1.279-3.868) | 0.005 |
| History of diuretics | 2.616 (1.387-4.934) | 0.003 |
| **Model 2 for MACEs** |  |  |
| SUA/Scr <3.45 | 2.340 (1.347-4.063) | 0.003 |
| Diuretics during hospitalization | 2.333 (1.293-4.210) | 0.005 |
| **Model 3 for Death** |  |  |
| SUA/Scr <3.45 | 2.228 (1.153-4.307) | 0.017 |
| History of diuretics | 3.614 (1.789-7.300) | < 0.001 |
| **Model 4 for Death** |  |  |
| SUA/Scr <3.45 | 2.391 (1.248-4.579) | 0.009 |
| Diuretics during hospitalization | 2.572 (1.243-5.324) | 0.011 |

Additional models were established to adjust potential confounders, especially the use of diuretics, by entering diuretic use and SUA/Scr into different Logistic regression models at a time. *All factors in each model were entered in the multivariate logistic regression analysis.

SUA/Scr, serum uric acid/serum creatinine; MACEs, major adverse cardiovascular events; 95% CI, 95% confidence interval
